# Enhanced Stability of Oral Vitamin C Delivery: A Novel Large-Scale Method for Liposomes Production and Encapsulation through Dynamic High-Pressure Microfluidization

**DOI:** 10.3390/nano14060516

**Published:** 2024-03-14

**Authors:** Eugenia Romano, Roberta Palladino, Mariagabriella Cannavale, Erwin Pavel Lamparelli, Barbara Maglione

**Affiliations:** 1 Farmaceutici Damor SpA, Via E. Scaglione 27, 80125 Napoli, NA, Italy; roberta.palladino@farmadamor.it (R.P.); gabriella.cannavale@farmadamor.it (M.C.); barbara.maglione@farmadamor.it (B.M.); 2 Department of Medicine, Surgery and Dentistry, University of Salerno, Via S. Allende, 84081 Baronissi, SA, Italy; elamparelli@unisa.it

**Keywords:** ascorbic acid, nanoencapsulation, high-pressure homogenizer, liposomes, drug delivery system, scale-up process, nanomaterial-based packages, oral supplements

## Abstract

In recent years, nanocarriers have been widely used as an effective solution for oral administration of pharmaceuticals. However, there is still an urgent need to speed up their translation to clinical practice. Cost-effective and industrially scalable methodologies are still needed. Herein, the production of vitamin C-loaded liposomes for nutraceutical purposes has been investigated and optimized by adopting a High-Pressure Homogenizer. Initially, the impact of process parameters on particles size, distributions, and morphology was explored. The findings document that the pressure and cycle manipulation allow for control over liposome size and polydispersity, reaching a maximum encapsulation efficiency exceeding 80%. This significantly improves the storage stability of vitamin C, as demonstrated by monitoring its antioxidant activity. Furthermore, the in vitro simulation of gastrointestinal digestion shows that liposomes could protect the active substance from damage and control its release in the gastrointestinal fluid. Thus, the whole nanodelivery system can contribute to enhancing vitamin C bioavailability. In conclusion, the results indicate that this innovative approach to producing vitamin C liposomes holds promise for clinical translation and industrial scale-up. Indeed, by utilizing food-grade materials and straightforward equipment, it is possible to produce stable and functional liposomes suitable for health products.

## 1. Introduction

During the last few decades, the increasing demand for products with high nutritional value has led to liposomes’ use as an oral delivery system by preserving and protecting sensitive materials in various food practices [1]. Liposomes are spherical-shaped nanostructures with a composition similar to that of cell membranes, consisting of one or more double phospholipid layers encapsulating an aqueous compartment [2]. The main advantages of their use as pharmaceutical carriers include biocompatibility, biodegradability, monodispersity, non-immunogenicity, and high capacity for entrapment of both hydrophilic and hydrophobic compounds. Thus, besides protecting bioactives during food processing or storage, liposomes delivery by oral administration can improve their bioavailability, achieving a controlled release and absorption to specific targets, especially molecules that are highly soluble in water [3]. Consequently, the stability and storage conditions of these nanocarriers loaded with nutraceuticals as vitamins and polyphenols, as well as their behavior in the gastro-intestinal transit, have attracted more and more attention [4,5].

For instance, Mohammadi et al. [6] reported the preparation of vitamin D-loaded liposomes for beverage fortification. Notably, they reached an encapsulation efficiency (EE) of 93%. However, sample stability was preserved only with storage in darkness at 4 °C. Sebaaly et al. [7] developed clove essential oil-loaded liposomes to protect eugenol from UV degradation and tested their stability for two months at 4 °C. Liposomes exhibited a steady nanometric size and an oligolamellar spherical structure with an EE of 72%. Similarly, Lee et al. [8] reported the preparation of multilamellar vesicles composed of soybean-derived L-a-phosphatidylcholine and 10% sterol (*w*/*w*). In this study, the nanovesicles were encapsulated with retinol, a hydrophobic compound that can easily be isomerized and photo-oxidized in a hydrophilic environment, resulting in activity loss. Notably, they obtained an EE higher than 99%. Furthermore, liposomes monitored at 4 and 25 °C, with and without light for up to 10 days, effectively protected the retinol from light- or heat-induced degradation.

Various approaches have been employed for liposomes encapsulation with vitamin C also known as ascorbic acid [9]. Vitamin C is an essential nutrient offering numerous benefits for human health [10]. It plays a crucial role in different physiological processes, including the collagen synthesis, antioxidant activity, and immune-modulating function [11,12]. Despite all these therapeutical properties, vitamin C cannot be autonomously synthesized by humans due to the lack of a specific enzyme, termed L-gulono-γ-lactone oxidase (GLO), in the biosynthetic pathway [13]. Moreover, its inherent instability and bad pharmacokinetic profile pose significant challenges for pharmaceutical and nutraceutical applications [14]. Indeed, factors such as exposure to light, heat, moisture, and oxidation can lead to rapid degradation, limiting its in vivo efficacy and shelf life [15].

To overcome all these challenges, nanoencapsulation has emerged as a promising approach to optimize its delivery through improved stability, controlled release, bioavailability, and protection against degradation [16].

Kirby [17] for the first time, described a dramatic increase in vitamin C liposomes’ antioxidant efficacy compared with a free vitamin C solution. More recently, Marsanasco et al. [18] prepared multilamellar SPC-based liposomes encapsulating vitamin C by the dehydration–rehydration method with an EE higher than 85%. Despite the promising results, the leakage % monitored at room temperature for 72 hours (h) was over 30%. Łukawski et al. [19] worked on a procedure free of harmful organic solvents. Liposomes were formulated by mixing glycerol-containing lipids and an aqueous solution of sodium ascorbate. Notably, a uniform population was formed spontaneously upon mixing with a final EE over 99%. Similarly, Chen et al. [20] developed a lipid-based formulation of vitamin C by processing food-grade materials with easily scalable equipment. Briefly, an aqueous solution of vitamin C was stirred along with glycerol at room temperature. Sunflower lecithin was then added to the mixture and blended at 3000 rpm. Interestingly, the researchers obtained unilamellar vesicles and a final EE of 43%. However, particles showed a broad size distribution, between 200 and 3000 nm.

Despite the extremely promising advantages offered by these nanovehicles, their production in the food and pharmaceutical industry is still challenging. Specifically, there are issues regarding mass recovery, including batch-to-batch irreproducibility, instability, and difficulties in scaling up the process [21].

Considering the recent advancements in the field, this study aims to investigate the effectiveness of vitamin C-loaded liposomes production through High-Pressure Homogenization (HPH). The High-Pressure Homogenizer has been extensively applied as a method for producing micro and nanoscale-size materials used in the pharmaceutical and food industry [22,23]. Among all the commercially available homogenizers, the Microfluidizer@ system presents some advantages for an efficient scale-up production of liposomes. Indeed, microfluidization combines the water jet, impinging stream, and traditional HPH technologies [24]. The microfluidizer primarily consists of a pump and a homogenizing valve. The pump propels the fluid through a narrow channel of either Y or Z-type, applying high pressure [25]. This leads to the acceleration of the suspension that is forced through a narrow gap. The generation of intense shear stress in a turbulent flow consequently prompts a separation of the mixture into little droplets with a precise size and a uniform distribution [26,27].

Herein, the formulation and process parameters were thoroughly investigated and optimized to ensure consistent results across batches, achieve an adequate particle size distribution (PSD), and obtain satisfactory mass recovery. Secondly, vitamin C was encapsulated within liposomes, and the resulting carriers were characterized in terms of particles size, zeta-potential, morphology, and EE%. Once the best operative parameters had been identified, a gold standard formulation was used for additional analysis of storage stability, antioxidant properties, and monitoring of vitamin C release in simulated gastro-intestinal fluids (GIF).

## 2. Materials and Methods

Soy lecithin was purchased from ABS^®^ FOOD (Vigonza, PD, Italy), vegetable origin refined glycerol was purchased from Spiga nord (Carasco, GE, Italy), sodium ascorbate was purchased from FARMALABOR (Canosa di Puglia, BT, Italy), and sorbitol was purchased from Roquette (Lestrem, France). All other chemicals were reagent-grade from Merck (Darmstadt, Germany) and were used without further purification steps unless otherwise specified.

### 2.1. HPH for Vitamin C-Loaded Liposomes Production

Vitamin C-loaded liposomes (Lipo-C) were prepared using an organic solvent-free procedure. Briefly, a coarse emulsion was obtained by dispersing lecithin 40% (*w*/*v*) into deionized water at 60 °C. Then, a 20% (*w*/*v*) glycerol concentration was added to the mixture under mild stirring. A solution of sodium ascorbate 37.5% (*w*/*v*) and sorbitol 5% (*w*/*v*) was added to the dispersion and mixed with an IKA, T 18 digital ULTRA-TURRAX^®^ 5000 rpm. The dispersion was homogenized using a bench-top LM20 Microfluidizer^®^ Materials Processor, Microfluidics, Westwood, MA, USA (94 × 71 × 56 mm, w × d × h). Three different pressure conditions were tested: 10,000, 20,000, and 30,000 PSI up to three cycles. The Y fixed-geometry interaction chamber (diamond F20 Y-75 μm chamber) of the microfluidic device drives the suspension into two opposite microchannels. Then, the two jets of liquid suspension are forced to collide with each other at high pressure in a turbolent flow. Temperature variations were monitored during HPH through a thermocouple at the inlet and outlet of the system. An ice bath to cool the cooling external coil was used to keep the temperature in a range from 4 to 10 °C. Optimum conditions have been selected for further investigation based on droplet size and particle size distribution.

### 2.2. Characterization of Lipo-C: Morphological, Physicochemical Analysis and Vitamin C Encapsulation Efficiency

To capture Transmission Electron Microscope (TEM) micrographs, 10 µl of each diluted sample were dropped on a Formvar/Carbon 200-mesh Cu Agar Scientific Ltd. (Ted Pella, Redding, CA, USA, Cat. No. 01800-F). Negative staining was then performed using a phosphotungstic acid solution 2% (*w*/*v*) directly made on the deposit for 60 s. Finally, the samples were air-dried overnight. The resulting images were obtained using bright-field mode (TEM mod. FEI TECNAI G2 200 kV S-TWIN equipped with a 4 K camera; electron source with LaB6 emitter; FEI Inc., Dawson Creek Drive, Hillsboro, OR, USA). Images were taken at 120 kV using a spot size of 3 and an integration time of 1 s. ImageJ software (version 1.54g for Windows) was used for the analysis and rescaling of each image.

Dynamic light scattering (DLS) was used to determine the liposomes’ size (Zeta sizer, Malvern Panalytical, Malvern, UK). The sample volume suitable for DLS analysis was 1 mL in a polystyrene 12 mm square glass cuvette with a square aperture for 90° sizing (Malvern; # PCS1115). Each sample was diluted 1:100 in Milli-q water and measured at 25 °C or 37 °C according to the aim of the analysis with a solid-state laser (λ = 633 nm) at a scattering angle of 173°. Zeta-potential measurements were also performed at a temperature of 25 °C on a Zetasizer Nano ZS (Malvern Panalytical, UK), loading the high-concentration surface zeta-potential cell with 1 mL of the 1:100 LNPs suspension. Zeta-potential charge values were derived from triplicate measurements, where each consisted of a minimum of ten individual runs.

To assess the EE% of vitamin C in Lipo-C, a purification step was performed to get rid of the excess of non-encapsulated molecules. Briefly, a membrane Corning^®^ Spin-X^®^ UF 6 mL Centrifugal Concentrator (10,000 MWCO Membrane) was centrifuged for 1 h at 4 °C 5000 rpm. The final amount of free vitamin C in solution was determined by UV-Vis Spectrophotometric analysis through a calibration curve at 240–265 nm (Figure S1). The EE% of vitamin C was calculated by Equation (1):

(1) $$\mathrm{EE}\%=\frac{\mathrm{Vt}}{\mathrm{Vi}\;}$$

where Vt is the total amount of vitamin C added in the suspension and Vi is the amount entrapped in the sample.

### 2.3. Stability Studies of Lipo-C

#### 2.3.1. Storage Stability by Size and Antioxidant Activity Monitoring

To investigate the storage stability of Lipo-C, freshly prepared samples sealed in glass tubes were stored at both 4 °C and 25 °C for 1 month in the dark and periodically checked for mean particle size, PDI, and vitamin C leakage. The Retention Rate (RR) was determined by measuring the encapsulated amount of vitamin C according to Equation (2):

(2) $$\mathrm{RR}\left[\%\right]=\frac{V\;\mathrm{after}\;\mathrm{Storage}}{\mathrm{Time}\;0\;V\;}$$

where V after storage is the total amount of vitamin C at a certain time point and Time 0 V is the vitamin C at time point 0.

The DPPH radical scavenging assay was determined as already reported in a previous work [28], with some slight modifications. Briefly, 0.5 mL of Lipo-C suspension was mixed with 1.5 mL of 0.016 mM DPPH ethanol solution. The mixture was kept in the dark for 30 min and then its absorbance was measured at 517 nm using a UV-vis spectrophotometer. The percentage of the DPPH-scavenging activity was calculated using Equation (3):

(3) $$\%\;\mathrm{Inhibition}\;=1-\frac{\left(A0-A1\right)}{A0}$$

where A0 is the absorbance of the control (DPPH without sample) and A1 is the absorbance of the test sample (the sample test and DPPH solution). Free vitamin C was used as positive control.

#### 2.3.2. Release Studies in Simulated GIF

Simulated gastric fluid (SGF) and simulated intestinal fluid (SIF) were prepared according to a previous study [29]. SGF consisted of sodium chloride (2 g/L), and pepsin (3.2 g/L). SIF consisted of calcium chloride (1.2 mM), sodium chloride (15 mM), pancreatin (4.76 g/L), and bile salts (5.16 g/L). The pH value was adjusted, respectively, to 1.2 and 6.8 using hydrochloric acid and sodium hydroxide. Next, 2.5 mL of Lipo-C was transferred into a 20 KDa cut-off dialysis tube immersed at 37 °C in SGF for 120 min and then in SIF for up to an additional 360 min. At proper time intervals, a certain amount of the dialyzed sample was withdrawn and replaced with an equal amount of PBS. The removed samples were analyzed spectrophotometrically. The cumulative amount of vitamin C released as a function of time was calculated using the following equation:

(4) $$\mathrm{Cumulative}\;\mathrm{Release}\;\left[\%\right]=\frac{\mathrm{Released}\;V}{\mathrm{Total}\;V\;}$$

#### 2.3.3. Statistical Analysis

All experiments were performed at least three times, and the data were expressed as the mean values ± standard deviation (SD). Before conducting the statistical analysis, the data underwent a normality check using Shapiro–Wilk’s test, which is the more appropriate method for small sample sizes (<50 samples). For this test, the null hypothesis assumes that the data are derived from a population with a normal distribution. Therefore, if the resulting *p*-value is above 0.05, the null hypothesis is accepted, indicating that the data are distributed normally. This is a prerequisite for performing the parametric tests, including Student’s *t*-test and ANOVA. The normality test confirmed that the data follow a normal distribution. Therefore, the statistical analysis involved the two-tailed independent Student’s *t*-test for comparing two independent groups or one-way ANOVA, followed by Dunnett’s multiple comparison test to examine differences among more than two independent groups. Significance was determined by accepting *p*-values less than 0.05 as significant. All statistical analyses were performed using GraphPad Prism software (version 6.0 for Windows). The findings were added to the graphs along with legends.

## 3. Results

### 3.1. Effect of HPH Operative Parameters on Lipo-C Preparation

This study aimed to investigate the effectiveness of a novel technology in the large-scale manufacturing of lipid-based nanocarriers loaded with vitamin C for nutraceutical applications. To achieve this, a Microfluidizer, i.e., a commonly utilized High-Pressure Homogenizer for the industrial-scale production of nanoemulsions, was employed.

The control of particle size and distribution during the microfluidization process is accomplished by manipulating operational parameters such as the valve’s geometry, pressure, and the number of cycles employed for suspending processing.

Herein, we chose a Y-type chamber with the minimum microchannels diameter (75 µm) to afford a reduction in particle size at the maximum deformation on suspension during nanoemulsion formation. Three pressure conditions (10,000-20,000-30,000 PSI) were tested for up to three cycles to investigate the effects of the High-Pressure Homogenizer operative parameters on Lipo-C structures.

**Figure 1:**
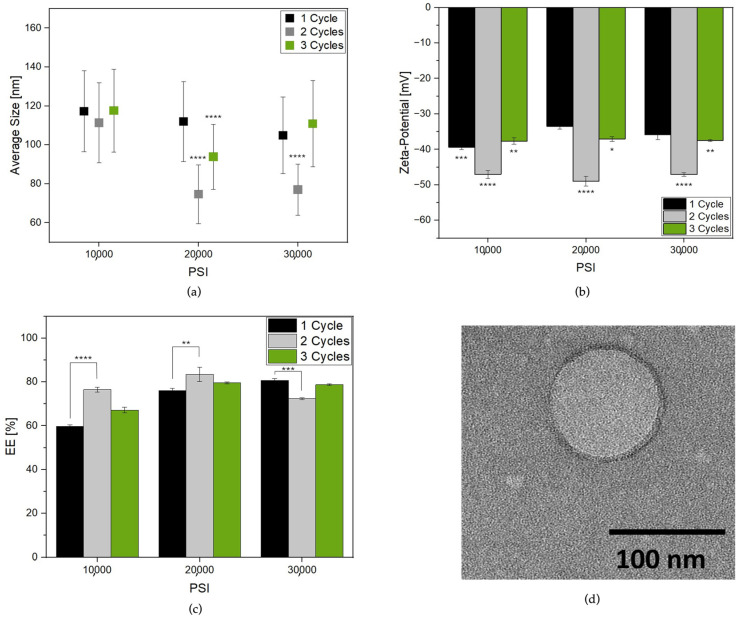
Characterization of Lipo-C formulations obtained by treatment at three different HPH pressures and cycles (**a**) particle average size; (**b**) zeta-potential; and (**c**) vitamin C EE%; (**d**) TEM image of Lipo-C obtained at optimal HPH process parameters (Lipo-C-20). The experiments were analyzed by one-way ANOVA, * *p* ≤ 0.05, ** *p* < 0.01, *** *p* < 0.001, and **** *p* ≤ 0.0001; n = 3.

Figure 1a reports the Lipo-C average size change from cycle to cycle at the selected PSI. The pre-homogenized sample presented a PDI of 0.456, indicating a very broad size distribution (Figure S2) which could be related to multiple lipid layers, fusion, or aggregation phenomena [5,23]. The HPH induced nanovesicle formation with a lower PDI and size distribution ranging from 50 to 140 nm. Three different phases were identified by increasing the number of cycles: HPH applied on the coarse emulsion at 10,000, 20,000 and 30,000 PSI for one cycle induced the formation of nanoparticles with lower polydispersity (Table S1). Then, the recirculation of samples in the chamber for two cycles allowed a further size and PDI reduction. Finally, at three cycles, a prompt increase in size and PDI, especially for the sample treated at 30,000 PSI, was observed. So, it can be speculated that aggregation and/or destabilization of particles occurred upon treatment [25].

To further corroborate our hypothesis, we reported the zeta-potential values for each sample. Indeed, the surface charge of the liposomes played a significant role in the stability of the system [30,31]. Interestingly, the zeta-potential (Figure 1b) became more negative when the samples were reprocessed for two cycles, while a slight increase was observed at one and three cycles, probably due to the system destabilization, in agreement with previous results.

Finally, an EE ranging from 59 to 83% was reported (Figure 1c). In agreement with the literature [25], Lipo-C HPH treatment for one cycle showed an EE increase as a function of pressure rise. Notably, this phenomenon was also visible for up to two cycles for samples treated at 10,000 and 20,000 PSI with a maximum EE of 83% for Lipo-C treated at 20,000 PSI for two cycles used as our “gold standard” (Lipo-C-20).

To gather insights into the morphological characteristics of our sample, TEM analysis was performed. In more detail, Figure 1d reports a TEM micrograph of Lipo-C-20. The image consistently revealed a spherical shape with a monolamellar bilayer and a size comparable to DLS measurement.

### 3.2. Lipo-C-20 Storage Stability and Behavior in Simulated GIF

In 2018, the FDA guideline for liposome drug products on an industrial scale underlined the necessity of stress stability tests to assess lipid nanoparticles’ size distribution, integrity, and to evaluate possible degradation due to hydrolysis of saturated and unsaturated lipids [32,33].

**Figure 2:**
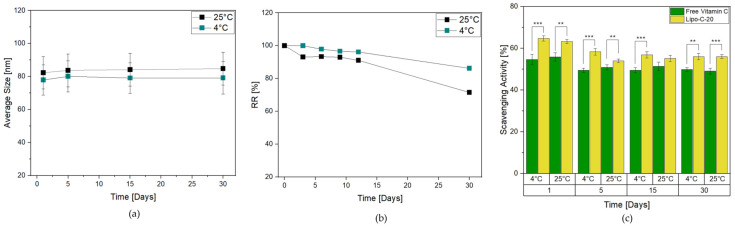
Effect of storage conditions of Lipo-C-20 over 1 month in the dark at 4 °C and 25 °C on (**a**) particles average size; (**b**) RR % of vitamin C; and (**c**) DPPH scavenging activity of Lipo-C-20 compared to a free vitamin C solution. The experiments were analyzed by two-tailed Student’s *t*-test, ** *p* < 0.01 and *** *p* < 0.001; n = 3.

Herein, Lipo-C-20 was stored at 4 °C and 25 °C in the dark for up to 1 month to evaluate its storage stability. It was subjected to a visual control to investigate the possible formation of mold or precipitation phenomena, as well as a DLS measurement. Lipo-C-20 kept at both 4 °C and 25 °C essentially retained the mean size and PDI values during the whole period examined, indicating the physical stability of the colloidal dispersions independently from the temperature (Figure 2a). Another important parameter for assessing the effectiveness of a transportation system is the RR, which is indicative of possible leakage phenomena during the storage. Interestingly, Figure 2b shows that vitamin C RR% of Lipo-C-20 kept at 4 °C over a period of 1 month did not exceed 8%. Conversely, a slight increase of 10% in RR reduction was observed at 25 °C. However, the results in both cases proved the chemical stability of the vitamin C and the very low tendency of its premature expulsion from our nanocarrier.

The antioxidant activity was then evaluated through the DPPH scavenging activity assay. The analysis of Lipo-C-20 compared to free vitamin C was performed in the dark at 4 °C and 25 °C (Figure 2c) considering the storage issues related to vitamin C degradation. The DPPH-radical scavenging rates of Lipo-C-20 were steady up to 55% over 1 month of storage independently from the temperature applied, suggesting that the incorporation of vitamin C into liposomes could considerably enhance its antioxidant property.

**Figure 3:**
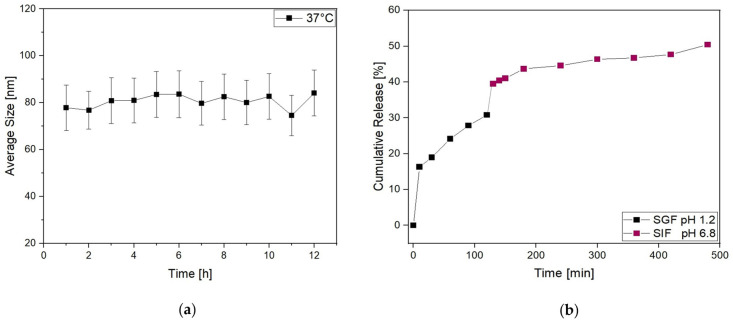
Physical stability of liposomes is expressed as (**a**) the average size and standard deviation of Lipo-C-20 observed after overnight incubation in PBS at 37 °C; (**b**) release profile of Lipo-C-20 in a simulated GIF expressed as cumulative release % of vitamin C from liposomes incubated 2 h in SGF pH 1.2 and then additional 6 h in SIF pH 6.8.

Then, to further investigate the behavior of Lipo-C-20 in the physiological environment, the size distribution was evaluated by DLS over time (up to 12 h) in PBS at 37 °C (Figure 3a). No significant increase in their average size and SD was observed. Thus, given our general purpose of overcoming the oral bioavailability challenges and therapeutic issues of vitamin C by a nanocarrier, the fate of Lipo-C-20 in GIF was investigated by an in vitro release study conducted at 37 °C up to 8 h under a mild and constant stirring (Figure 3b). An initial burst effect with a release of up to 30% during the first 120 min in the acid medium was observed. This may be related to the immediate release of the unencapsulated and surface-associated vitamin C. However, the vitamin C embedding into liposomes slowed down the dialysis bag release compared to free vitamin C (Figure S3). The chyme obtained from the first 120 min in the SGF was then displaced in the SIF. In the intestinal stage, a considerable release was observed with a final vitamin C cumulative release rate of about 50% following 8 h of gastrointestinal digestion.

## 4. Discussion

Vitamin C is a commonly used ingredient in nutraceutical and pharmaceutical products due to its powerful properties. It has long been recognized for supporting endothelial cells and participating in various critical functions within the vascular system. These functions include promoting the synthesis and deposition of type IV collagen connective tissue and protein fibers in the basement membrane, which provide strength to teeth, gums, muscles, blood vessels, and skin and prevent apoptosis of endothelial cells [34]. Also, it plays an important role in the immune system by aiding white blood cells in fighting infections and facilitating iron absorption within the body [11,35]. However, since it is highly soluble in water, vitamin C cannot be stored in the body and requires a daily intake [36]. Furthermore, its therapeutic use has also been hindered by the difficulty of reaching an effective blood concentration without administering it at very high doses that are burdened with significant side effects. These side effects include an increased risk of hemolytic anemia in patients with G6PD (Glucose-6-phosphate dehydrogenase) deficiency, potential renal failure, and the formation of kidney stones due to the acidification of urine caused by vitamin C. Additionally, the metabolism of vitamin C can result in the formation of oxalic acid, potentially leading to hyperoxaluria [37]. Concerning pharmacokinetic profile, once vitamin C has been orally administered, its absorption in humans is remarkably intricate and differs from those of most low-molecular-weight compounds. As a result of the non-linear uptake kinetics of vitamin C, plasma concentrations tend to reach saturation at around 70 µmol/L. This saturation point is typically achieved with oral intakes ranging from 200 to 400 mg/day [38]. Indeed, once plasma vitamin C concentrations reach saturation, the additional amount is excreted.

As stated in the introduction, the use of lipid-based nanocarriers for the oral delivery of vitamin C can protect its activity, enhancing its in vivo pharmacokinetic profile and stability over time. Despite the growing number of investigations in the field, essential acknowledgments of the main pathways of these nanocarriers are still missed. Consequently, their development and market viability for clinical use still remain challenging.

According to the NCBI, about 70,000 investigations have been conducted on the uses of vitamin C; however, less than 3% concerns vitamin C nanodelivery [39]. Furthermore, this limited percentage is mostly focused on the improved bioavailability by liposomal formulations, and only a few clinical trials have been dedicated to its therapeutic application (NCT06044194, NCT04947488, NCT05412160).

Regarding the market, liposomal vitamin C oral supplements are available mainly as tablets. Even though a liquid formulation could improve patient compliance in oral administration, nutraceutical products with liposomal suspensions are still very few and the supplement of the maximum recommended dosage per day requires a high amount of product, between 5 and 10 mL. This is mainly due to low EE, storage instability of the final batches monitored over time, and an unsatisfactory batch-to-batch reliability.

To address these needs, herein, we developed a large-scale and organic solvent-free method to product a liquid formulation of vitamin C-loaded liposomes using food-grade ingredients.

Herein, we selected a lecithin powder from NON-GMO soybeans. In more detail, this natural mixture of phospholipid provides phosphatidylcholine, the most widely applied phospholipid for liposome formulation, in a concentration ranging between 19.5 and 22.5% [40,41]. Also, the presence of two unsaturated acyl chains gives a higher permeability to the lipidic matrix, allowing the adsorption of the active substance [42].

To develop a harmful solvent-free method for liposome production, glycerol has been used to solubilize the lecithin powder. Indeed, unlike other organic solvents such as ethanol, it does not require precautions for safe handling and storage. Furthermore, its vegetable origin and extensive application as a food additive make it suitable for our purpose. Indeed, the proper application of bioactive-loaded liposomes in the nutraceutical industry should involve the use of excipient materials and bioactives recognized as safe.

As for the downstream processing, we investigated HPH using a microfluidizer for liposome formulation. One of the main advantages of this platform is that nanoparticle physical properties, including size distribution and morphological stability, can be controlled by varying the pressure and the number of cycles applied in a homogenizing chamber [43].

The two main interaction chambers used are the “Y type” and “Z type”, which differ in terms of geometry, minimum internal dimension, and final applications [44]. While the Z-type chamber is usually applied for cell disruption, the Y channel is ideal for particles formation and encapsulation in liquid-to-liquid suspensions [45]. Herein, we took advantage of the Y-type chamber with the minimum microchannel dimension (model F20 Y-75 μm chamber). This results in the passage of coarse emulsion through a 75 μm orifice, leading to a consistent particle size reduction and uniform dispersion of the liposomes [43].

During the processing three homogenizing pressures were tested (10,000-20,000-30,000 PSI) up to three cycles.

As expected, increasing the pressure and the number of cycles led to a decreased mean particle size and PDI. This phenomenon is ascribed to the high stress forces along the cavitation, shear, and impact generated in the microchannels. Notably the lowest mean particle size was obtained after treatment at 20,000 PSI for two cycles, while a prompt increase in particle dimension and PDI was visible after three cycles.

Thus, we speculate that instability phenomena such as the disruption and/or aggregation of particles occur due to overprocessing of samples, as stated by others [43,46]. Our hypothesis was also confirmed by changes in the surface charge of samples. Generally, a colloidal system would be stable if the absolute value of the zeta-potential is above 30 mV [47]. So, the progressive tendency of zeta-potential to come close to −50 mV for up to two cycles indicated an improvement in the liposome’s physical stability that reduced after the treatment at one and three cycles [48]. In addition, the EE was higher after two cycles independently from the applied pressure, reaching the maximum at 20,000 PSI.

Taking all these achievements together, we selected the best operative condition and proceeded with a preliminarily in vitro test on the oral behavior of the gold standard formulation Lipo-C-20.

Another important aspect to be considered in the engineering of a nutraceutical nanodelivery system is its passage once it has been orally administered to patients.

The expected release site of ascorbic acid is the small intestine rather than the stomach. Thus, the main goal for an ideal oral formulation of vitamin C is the transport to the intestinal epithelia for absorption and metabolization [49].

Several studies reported on the application of polymeric, liposomal, and micellar micro and nano-carriers to improve vitamin C bioavailability [39]. As an example, gelatin-coated microcapsules were encapsulated with ascorbic acid with EE of about 94%, but the release of ascorbic acid in the stomach was faster than in the intestine [50]. Microencapsulation of vitamin C for its oral delivery has also been provided using chitosan, a hydrophilic polysaccharide of low toxicity that can transport active compound through the gastro-intestinal tract thanks to its mucoadhesive characteristics [51]. Spherical, stable chitosan particles can be obtained with the use of cross-linking agents and organic solvents; however its low solubility at physiological pH limits its application. Indeed, vitamin C-loaded chitosan nanoparticles (VC-CS) showed a fast release profile in PBS [50,52]. Increased protection of the payload was obtained by a double layer coating. For instance, Dong et al. entrapped vitamin C into zein/chitosan nanoparticles of spherical shape and with particle size between 720 and 1100 nm [53]. Notably, the vitamin C release rate in the gastric environment was about 5% compared to 20% for VC-CS.

In another study, encapsulation of vitamin C was obtained through electrostatic interaction between glycidyl trimethyl ammonium chloride chitosan and phosphorylated-cellulose nanocrystals. Interestingly, the EE was 90% while the digestion simulation at 8 h achieved a release of approximately 27% [54]. However, considering industrial production, some points need to be addressed regarding the time-consuming steps of production and purification, as well as the use of cost-effective materials.

Regarding liposomes, they offer numerous benefits derived from their unique architecture. The lipid bilayer surrounds an aqueous inner core along with water-soluble compounds, and this results in the protection of active substances from hostile digestive conditions [3]. Indeed, our work demonstrated that liposomes are capable of both protecting vitamin C in acid conditions and releasing it in the intestinal fluids.

Liposomes’ fate after oral administration is still unclear; however, once absorbed as integral vesicles via the M cell-to-lymph pathway in the small intestine [55], the spherical cell membrane–like lipid bilayers can better adhere to biomembranes compared to other nanomaterials, resulting into a fusion of the lipidic portion with the cellular membrane and then a selective release of the active compound in the targeted area.

In this regard, the size of nanoparticles is another crucial factor to consider. Indeed, several studies reported that clearance is inversely related to size and small unilamellar liposomes and micronized emulsions circulating the blood for the longest amount of time. Thus, small liposomes with a mean size lower than 200 nm, such as our Lipo-C-20, may be drastically more efficient at intracellular delivery of encapsulated compounds. However, further investigation in the field is needed.

## 5. Conclusions

Homogenous, stable, nanometric-sized, and monolamellar liposomes with a high loading rate over 80% were obtained from processing samples by HPH at 20,000 PSI for two cycles (Lipo-C-20). Once operative conditions have been optimized, the physicochemical stability of Lipo-C-20 was monitored for up to 1 month of storage at both 4 °C and 25 °C. The results suggested that the encapsulation of vitamin C could significantly improve the storage stability compared to free vitamin C in solution. No leaking effects were observed, demonstrating the payload of the active substance. Furthermore, the release of vitamin C was monitored in a simulated GIF for up to 8 h.

Notably, vitamin C is released slowly in SGF with a final concentration no higher than 30% against the 70% reached by the free vitamin C in solution. This may be related to the immediate release of the unencapsulated and surface-associated vitamin C. However, over 50% of the active substance is then released in SIF, where the absorption is desirable.

In conclusion, this novel large-scale method allows food-grade materials use and non-harmful solvents to produce vitamin C-loaded liposomes. The encapsulation of this active ingredient into our nanocarrier enhanced its stability and protection from external factors, as demonstrated by the storage stability and antioxidant activity studies. Overall, the liposomal formulation ensures a thorough and prolonged release of the active compound following oral administration, leading to enhanced therapeutic effectiveness.

## Data Availability

Data are contained within the article.

## Supplementary Materials

The following supporting information can be downloaded at https://www.mdpi.com/article/10.3390/nano14060516/s1, Figure S1: Calibration curve of free vitamin C; Figure S2: particle size distribution by DLS measurement of coarse emulsion obtained before HPH treatment; Table S1: HPH overall process parameters and their effect on particle size, PDI, EE%, and zeta-potential of Lipo-C formulations; Figure S3: cumulative release % of free vitamin C from a dialysis bag in GIF up to 8 h.
