# Supplementary material for: Enhanced Stability of Oral Vitamin C Delivery: A Novel Large-Scale Method for Liposomes Production and Encapsulation through Dynamic High-Pressure Microfluidization
Source: Nanomaterials (Basel). 2024 Mar 14;14(6):516. doi: 10.3390/nano14060516 (PMC10975307; doi:10.3390/nano14060516)
Supplement: Supplementary file 1 [file nanomaterials-14-00516-s001.zip › nanomaterials-2896585-supplementary.pdf]

Supporting Data

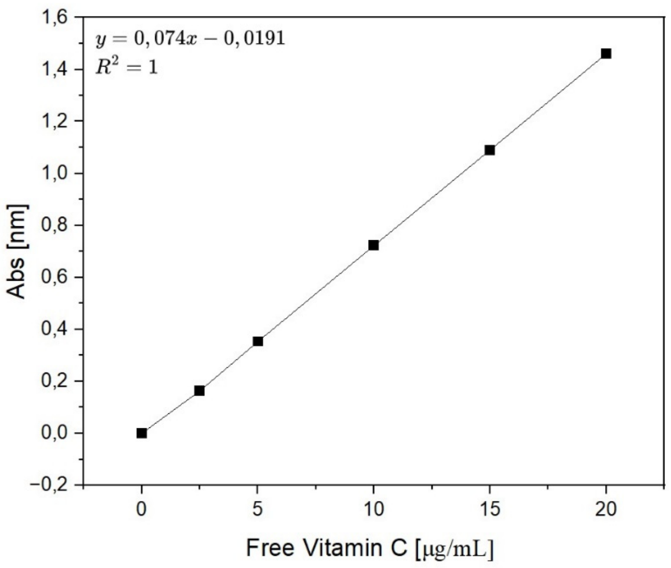

Figure S1. Calibration curve of Free Vitamin C. UV-Vis Spectrophotometric analysis was performed at 265 nm with a Vitamin C concentration in the range 2.5-20 μg/mL.

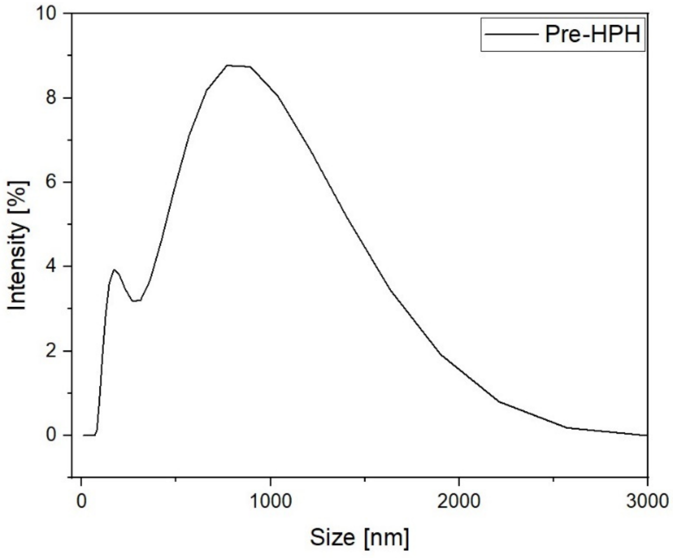

Figure S2. Particle Size Distribution by DLS measurement of Coarse emulsion obtained before HPH treatment.

Cycles	Pressure (PSI)	Diameter (nm)	PDI	EE (%)	Zeta-Potential (mV)
1	10000	117.277	0.252	59.727	-39.393 ±0.657
	20000	112.003	0.270	76.031	-33.480 ± 0.824
	30000	104.852	0.282	80.68	-35.887 ± 1.315
2	10000	111.338	0.272	76.533	-47.04 ±1.130
	20000	81.692	0.248	83.479	-48.97±1.350
	30000	76.987	0.233	72.366	-47.06±0.480
3	10000	117.575	0.262	67.178	-37.657± 0,900
	20000	93.861	0.253	79.597	-37.083± 0.695
	30000	110.906	0.320	78.769	-37.470 ± 0.269

Table S1. HPH overall process parameters and their effect on particle size, PDI, EE% and Zeta-potential of Lipo-C formulations.

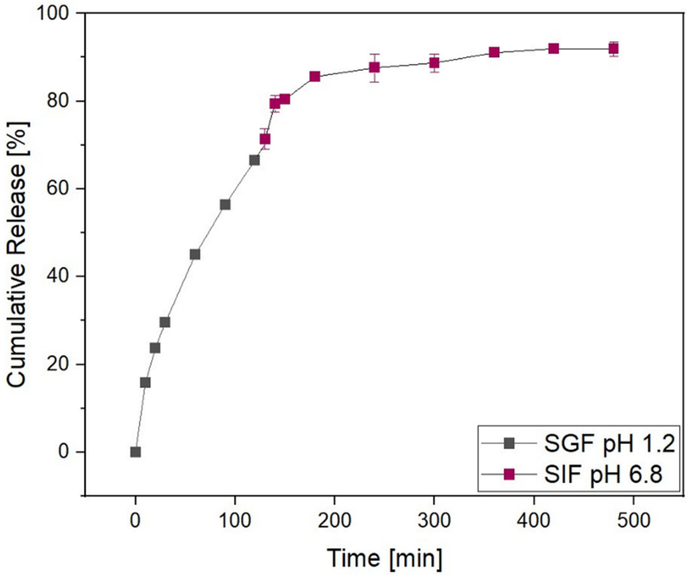

Figure S3. Cumulative Release % of Free Vitamin C from a dialysis bag in GIF up to 8 h.
